# The nature of ligand efficiency

**DOI:** 10.1186/s13321-019-0330-2

**Published:** 2019-01-31

**Authors:** Peter W. Kenny

**Affiliations:** Berwick-on-Sea, North Coast Road, Blanchisseuse, Saint George Trinidad and Tobago

**Keywords:** Drug design, Fragment-based lead discovery, Group efficiency, Ligand efficiency, Maximal binding affinity, Molecular interactions, Molecular recognition, Property-based design, Structure–activity relationship, Thermodynamics

## Abstract

Ligand efficiency is a widely used design parameter in drug discovery. It is calculated by scaling affinity by molecular size and has a nontrivial dependency on the concentration unit used to express affinity that stems from the inability of the logarithm function to take dimensioned arguments. Consequently, perception of efficiency varies with the choice of concentration unit and it is argued that the ligand efficiency metric is not physically meaningful nor should it be considered to be a metric. The dependence of ligand efficiency on the concentration unit can be eliminated by defining efficiency in terms of sensitivity of affinity to molecular size and this is illustrated with reference to fragment-to-lead optimizations. Group efficiency and fit quality are also examined in detail from a physicochemical perspective. The importance of examining relationships between affinity and molecular size directly is stressed throughout this study and an alternative to ligand efficiency for normalization of affinity with respect to molecular size is presented.
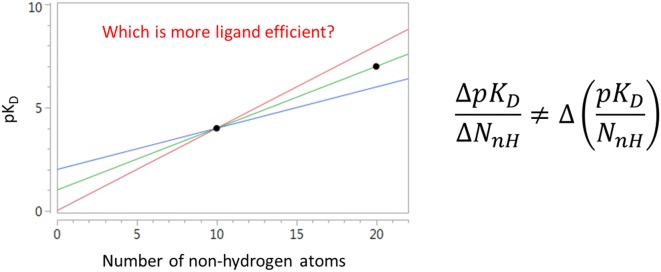

## Introduction

“I know I could,” the VP of Discovery responded tartly. “But what do you think you’re here for? I could order my own consumables, too, but that’s Milo’s job. Your job is to lead us in prayers, and from now on you’re going to lead us in a prayer for more ligand efficiency in every project. Is that clear? I think more ligand efficiency is something really worth praying for”.

Adapted from Joseph Heller, Catch 22

Ligand efficiency (LE) is, in essence, a good concept that is poorly served by a bad metric. It was introduced [1] as *“a useful metric for lead selection”*, has been discussed at length in reviews [2–6] and is routinely tracked in drug discovery projects. There are actually two ligand efficiencies in drug discovery and these can be seen as different manifestations of what might be called molecular size efficiency (MSE). First, the LE concept, sometimes summarized as ‘bang for buck’, which can be expressed in terms of the sensitivity of affinity to an increase in molecular size. Second, the compound-level LE metric (more accurately, family of metrics) that was introduced [1] with a view to normalizing affinity of compounds with respect to molecular size. While the LE concept has a solid basis, the LE metric cannot be regarded as physically meaningful because perception of efficiency varies with the concentration unit in which affinity is expressed [7, 8]. The difficulty stems from the inability of the logarithm function to take a dimensioned [9] argument which means that it is necessary to scale a K_D_ value by an arbitrary concentration unit to enable its logarithm to be calculated.

Drug design is incremental in nature. This reflects a view [10–12] that it is easier to understand and predict differences in chemical behavior between structurally-related compounds than it is to make absolute predictions directly from molecular structure. Drug action is driven by concentration and affinity determines sensitivity of response to this driving force. Drug design is a multi-objective [13] endeavor and some objectives, such as maximization of affinity against the therapeutic target(s) and minimization of affinity against anti-targets, can be defined clearly. Other objectives, such as controllability of exposure are much more difficult to define and this means that drug design is typically indirect. One significant difficulty [14] in drug design is that unbound intracellular concentration [15, 16] cannot generally be measured for drugs in vivo.

Most chemical starting points for design lack the affinity required to function as drugs and optimization typically results in increased lipophilicity, molecular size and molecular complexity [17, 18]. This is the essence of lead-likeness [19]. The Rule of 5 (Ro5) [20] highlights excessive molecular size and lipophilicity as primary design risk factors. Risks associated with molecular complexity [18] are more likely to be encountered in the screening phase of a project. Molecular complexity can also be seen inversely as the degree to which a compound is structurally prototypical [21, 22] (e.g., minimally substituted) and might also be defined in terms of molecular shape [23] or roughness [24, 25] of the molecular surface. Molecular recognition [26] provides much of the conceptual framework for drug design and many medicinal chemists consider molecular interactions [27] when elaborating chemical start points. While a structure–activity relationship (SAR) can point to the importance of individual interactions, the contribution of a protein–ligand contact to affinity is not, in general, an experimental observable [8, 28].

In property-based design [29, 30], risks associated with structural elaboration, such as poor oral absorption, are assessed according to physicochemical criteria. Within this framework, the most efficient optimization paths are those for which the necessary potency gains are accompanied by the smallest increases in perceived risk. One general objective of optimization projects has been stated [31] as *“ensuring that any additional molecular weight and lipophilicity also produces an acceptable increase in affinity”.* Efficiency can be seen as sensitivity of affinity to increased risk and this is the basis of what might be termed the LE concept. Kuntz et al. [32] examined the response of maximal affinity to number of non-hydrogen atoms and Hajduk [33] noted that *“along the path of ideal optimization, an increase of 1 pK*_*D*_ *unit can be expected for every 64 mass units”*. Saxty et al. [34] defined group efficiency (GE) for substitutions by scaling the change in affinity resulting from addition of a substituent by the number of non-hydrogen atoms added. The idea of quantifying sensitivity of chemical behavior to changes in molecular structure can be traced to the work of Hammett [35, 36] and the activity cliff [37, 38] concept can be seen as part of the same general framework.

Compound-level efficiency metrics are typically constructed by either scaling (i.e., divide affinity by risk factor) or offsetting (i.e., subtract risk factor from affinity) [8]. LE was introduced [1] as a metric to normalize affinity with respect to molecular size by scaling the standard free energy of binding, ΔG°, by the number, N_nH_, of non-hydrogen atoms (the term heavy atoms is also used) in the molecular structure as follows:

1 $$ \Delta g\left( {T,P,C{^\circ }} \right) = - \left( {\frac{{\Delta G^\circ }}{{N_{nH} }}} \right) $$

The standard state was not specified when the LE metric was introduced [1] although it appears to be widely believed [3] that C° must be set to 1 M for calculation of LE. The Achilles heel of the LE metric is its nontrivial dependency [7] on C° and, as conventionally [3] defined, LE has a 1 M concentration unit built into it. As noted by Gilson et al. [39], the choice of a particular value of C°, such as 1 M, to define the standard state is entirely arbitrary and a requirement that C° only take a specific value cannot be accommodated within the framework of thermodynamics. This means that LE cannot be defined objectively in absolute terms for individual compounds because there is no physical basis for favoring a particular value of C° for calculation of LE.

Other measures of molecular size can be used and binding efficiency index (BEI) [2] has been defined as a dimensionless quantity by scaling pIC_50_ by the numerical value of molecular weight (MW) expressed in kDa:

2 $$ BEI = \frac{{ - \log_{10} \left( {IC_{50} /M} \right)}}{{\left( {MW/kDa} \right)}} $$

Equation (2) shows that, like the conventional definition of LE, BEI has a concentration built into it because IC_50_ has to be divided by 1 M so that the logarithm can be calculated. It has been suggested that BEI should be interpreted as *“binding per gram”* (of ligand) [40]. However, binding per gram is identical to binding per mole even though the numerical values of the two quantities differ. Molecular recognition [26, 27] can be seen as a process in which molecules present their surfaces to each other and molecular surface area is, arguably, the most relevant measure of molecular size when examining affinity and potency data. Molecular surface area and molecular volume both vary with conformation and this complicates the use of these properties as molecular size measures in drug discovery. It should be stressed that the difficulties stemming from the arbitrary nature of C° (and the 1 M concentration unit used to express potency) cannot be addressed by simply using a different measure of molecular size such as molecular weight [2] or molar mass [40] for scaling affinity. A corollary of this is that LE, BEI and related metrics cannot be used to address the question of which measure of molecular size is most appropriate for drug design. In any case, the different measures of molecular size are likely to be highly correlated.

Although a quantity derived by scaling ΔG° by a risk factor does not have physical significance, offsetting affinity by a risk factor may give a physically meaningful quantity [8]. Provided that ligand ionization is insignificant, ligand lipophilicity efficiency (LLE) [41], which is also known as lipophilic ligand efficiency (LLE) [3] and lipophilic efficiency (LipE) [42], can be interpreted as the ease of transfer of a ligand from 1-octanol to its binding site [8]. Furthermore, some of the limitations of the 1-octanol/water partitioning system become less significant when working within structural series, as is usually the case for lead optimization [43]. While physical interpretability is certainly a desirable feature for a drug design metric, this alone does not guarantee that a metric will be usefully predictive in drug design.

The principal objectives of this study are to provide an in-depth analysis of LE (and its variants) and to highlight ways in which consideration of LE as a concept might address the serious deficiencies of the compound-level metric. LE is discussed in terms of molecular interactions and binding thermodynamics and some of this discussion is likely to be generally relevant to drug design. A recurring theme in this study is a view that it is generally better to observe the response of affinity to molecular size directly rather than through the distorting lens of a flawed LE metric.

## Molecular size and design risk

It is important that drug discovery scientists be fully aware of the assumptions on which the LE metric is based and that they carefully consider their motivation for using LE (or indeed any design guidelines). Property-based design [29, 30] can be seen in terms of balancing the risk associated with poor physicochemical characteristics against the risk of not being able to achieve the necessary level of affinity. Ro5 [20] is based on analysis of property distributions of drugs (defined as compounds that had progressed into Phase 2 trials) and the assessment of risk is indirect because non-drugs were not included in the original analysis. Ro5 [20] neither takes account of correlations between risk factors nor does it provide a means to deconvolute the risks associated with excessive molecular size and lipophilicity. The LE metric can be seen as a simple means with which to balance risk and there are more rigorous and sophisticated ways for doing this [44]. Simple drug design guidelines based on molecular size and/or lipophilicity typically become progressively less useful as more measured data become available to the drug discovery team.

Drug design guidelines are typically based on trends observed in data and the strengths of these trends indicate how rigidly guidelines should be adhered to. While excessive molecular size and lipophilicity are widely accepted as primary risk factors in design, it is unclear how directly predictive they are of more tangible risks such as poor oral absorption, inadequate intracellular exposure and rapid turnover by metabolic enzymes. This is an important consideration because the strength of the rationale for using LE depends on the degree to which molecular size is predictive of risk. Drug discovery scientists need to be wary of correlation inflation [14] (the term voodoo correlation [45] is also used) which can be loosely defined as presentation or analysis of data in any way that makes trends appear to be stronger than they actually are. Correlation inflation is a particular concern when analysis of proprietary data is presented in support of a view that a set of guidelines is especially useful or predictive. Published analyses of relationships [41, 46] between pharmacological promiscuity and molecular size and lipophilicity exemplify the problem. Comparison of average values without taking account of variance is one way in which trends can be made to appear to be stronger than they actually are and correlation inflation is acknowledged [47, 48] as an issue in drug design. Variance in the dependent variable can also be hidden by representing a distribution (e.g., aqueous solubility of compounds with property forecast index in the range 6–7) by a single percentile (e.g., percentage of those compounds with aqueous solubility > 200 µM) [49].

The relevance of data must also be considered when using physicochemical characteristics such as molecular size to assess risk. For example, an activity threshold [41] of > 30% inhibition at 10 µM for promiscuity analysis is not especially relevant if considering the likelihood of off-target effects for a drug with a peak unbound plasma concentration of 100 nM. Sample bias can be significant, even in large datasets, as exemplified by divergent conclusions of two apparently similar studies [41, 46] with respect to the relationship between pharmacological promiscuity and molecular size. The observation that average molecular weight appears to decrease [46] with promiscuity is particularly relevant to the use of LE because promiscuity would generally be considered [41] to be an undesirable characteristic for a compound. Drug designers should not automatically assume that conclusions drawn from analysis of large, structurally-diverse data sets are necessarily relevant to the specific drug design projects on which they are working.

## Thermodynamic aspects of ligand–protein association

The LE metric [1] was introduced in thermodynamic terms and it is sometimes believed that it measures the degree to which molecular interactions between ligand and target are optimal. For example, it has been asserted [50] *“Because of these optimal interactions, fragments are very ‘atom efficient’ binders, demonstrated by high ligand efficiency”*. This section focuses on thermodynamic [7, 39] aspects of protein–ligand association most relevant to LE and to the interpretation of affinity in terms of molecular interactions [26, 27].

The standard free energy of binding, ΔG°, [39] can be written in terms of the gas constant (R), thermodynamic temperature (T), C° and the equilibrium concentrations of protein ([P]), ligand ([L]), and protein–ligand complex ([P·L]):

3 $$\Delta G^\circ = RT\ln \left( {\frac{\left[ P \right]\left[ L \right]}{{\left[ {P \cdot L} \right]C^\circ }}} \right) $$

Equation (3) shows that ΔG° is a function of C° and this is one reason that values of standard free energy of binding should not be termed absolute. By convention, C° is taken to be 1 M although this is arbitrary and the value of C° has no physical significance [39]. In thermodynamic analysis, a change in perception resulting from a change in a standard state definition would generally be regarded as a serious error rather than a penetrating insight. In some situations, the dissociation constant, K_D_, is defined to be equal to the argument of the logarithm in Eq. (3) and is therefore dimensionless. However, in medicinal chemistry, biochemistry and biophysics, K_D_ values are conventionally quoted in units of concentration and Eq. (3) can be written as:

4 $$\Delta G^\circ \left( {T,P,C^\circ } \right) = RT\ln \left( {\frac{{K_{D} \left( {T,P} \right)}}{C^\circ }} \right) $$

Equation (4) shows that a tenfold increase in C° leads to a decrease in ΔG° of 1.36 kcal/mol at 298 K. The sign of ΔG° has no special significance and simply indicates whether or not K_D_ is greater or less than C°. The dependence of ΔG° on C° is a consequence of the stoichiometry of association of ligand with target and ΔG° for formation of a ternary complex (relevant when considering the thermodynamic consequences of fragment linking) will exhibit a different dependence on C° to ΔG° for a binary complex. The stoichiometry corresponding to a ΔG° value is specified by the change, ΔN, in the number of species for the corresponding reaction and it can also be seen as a ‘hidden dimension’ of ΔG°. For example, formation and dissociation of 1:1 complexes have ΔN values of − 1 and + 1 respectively. The value of ΔN determines the dimensions of the corresponding equilibrium constant:

5 $$ \dim K = \left( {concentration} \right)^{\Delta N} $$

The dependence of ΔG° on C° is a consequence of the loss of translational entropy resulting from association and it has two important implications. First, ratios of ΔG° values also depend on C° even though the ratios themselves are dimensionless and ΔG° values should therefore be compared as differences (i.e., ΔΔG). Second, if a free energy change is written as a sum of free energy changes then the sum needs to have the same dependency on C° as the original free energy change since the equality must hold for all values of C°. This is equivalent to requiring that the sum of ΔN values for the components of a free energy decomposition be equal to the ΔN value for the free energy change that is decomposed.

One way in which stoichiometry can be accounted for in free energy decompositions is to associate each free energy change with its corresponding ΔN value using square brackets. The study by Jencks [51] on attribution and additivity of binding energies can be used to illustrate this. Jencks [51] defined the intrinsic binding energy for a group X as the difference in ΔG° for compounds in which X is present (AX) or absent (A) in the relevant molecular structures:

6 $$ \Delta G_{X}^{i} \left[ 0 \right] = \Delta G_{AX}^{\circ } \left[ { - 1} \right] - \Delta G_{A}^{\circ } \left[ { - 1} \right] $$

The intrinsic binding energy is associated with a zero value of ΔN and is therefore independent of C°. Jencks [51] writes the ΔG° value for a compound with linked groups A and B in its molecular structure as the sum of the intrinsic binding energies of A and B, and the “connection Gibbs energy” (ΔG^s^):

7 $$ \Delta G_{AB}^{\circ } \left[ { - 1} \right] = \Delta G_{A}^{i} \left[ 0 \right] + \Delta G_{B}^{i} \left[ 0 \right] + \Delta G^{s} \left[ { - 1} \right] $$

Equation (7) is particularly relevant to fragment linking and it is important to note that ΔG^s^ does depend on C° [7, 51]. In some studies, ΔG° is decomposed into a value corresponding to zero molecular size (ΔG_MS = 0_) and a ΔΔG value:

8 $$ \Delta G^\circ \left[ { - 1} \right] = \Delta G_{MS = 0} \left[ { - 1} \right]\,+\,\Delta \Delta G\left[ 0 \right] $$

For example, Kuntz et al. [32] and Saxty et al. [34] used ΔG_MS = 0_ values of 0 and + 4.2 kcal/mol respectively. While this decomposition is valid to the extent that the sum has same dependence on C° as ΔG°, the assignment of an affinity value (e.g., K_D_ = 1 M) to a solute of zero molecular size for efficiency calculations does not appear to have any physical basis.

One general approach to modelling affinity is to use Eq. (9) in which A_i_ (i > 0) is a parameter associated with the substructure i and n_i_ is the number of occurrences of that substructural element:

9 $$ \Delta G^\circ \left[ { - 1} \right] = A_{0} \left[ { - 1} \right] + \mathop \sum \limits_{i = 1}^{{N_{SS} }} n_{i} \times A_{i} \left[ 0 \right] $$

The A_0_ term has the same dependency on C° as ΔG° and its inclusion in Eq. (9) allows changes in concentration unit to be easily accounted for. In Free-Wilson analysis [52] the substructures are typically groups at substitution sites on a scaffold, the n_i_ values are either 1 or 0 and A_0_ may correspond to the affinity of the unsubstituted scaffold. In the analysis of functional group contributions by Andrews et al. [53], A_0_ corresponds to the entropy term which was set to − 14 kcal/mol rather than derived by fitting the data. Schemes for decomposition of ΔG° based on Eq. (9) cannot be considered to be group additive because of the presence of the A_0_ term which is not associated with any group.

It has been asserted [54] that *“Ligand efficiency can be recast as a special case of group additivity where ΔG/HA is the group equivalent”* but this does not properly account for the stoichiometry of the binding. Unlike in Eq. (9), there is no is A_0_ term when ΔG° (ΔN = − 1) is decomposed into a sum of N_nH_ equal atom-based terms and this leads to significant difficulties. Specifically, each term in the sum must have an identical dependence on C° while the sum of terms needs to reproduce the dependence of ΔG° on C°. While this can be achieved algebraically by assigning a fractional stoichiometry to each atom-based term, the physical meaning of the resulting atom-based terms remains obscure. For example, the numerical values that result from dividing ΔG° values of 5 kcal/mol and 10 kcal/mol by 10 and 20 respectively are identical. However, the two quantities cannot be equated because they differ in their dependence on C°. In contrast, the enthalpy of binding, ΔH, does not depend on C° and so enthalpic efficiency [55] can be defined unambiguously.

An equivalent way to examine the stoichiometry issue is to consider the implications of writing K_D_ as follows where k_nH_ corresponds to Δg as defined in Eq. (1):

10 $$ K_{D} = \left( {k_{nH} } \right)^{{N_{nH} }} $$

Consider two compounds X (K_D_ = 10^−3^ M; N_nH_ = 10) and Y (K_D_ = 10^−6^ M; N_nH_ = 20) that would usually be considered to be equally ligand-efficient (Δg = 0.4 kcal/mol per non-hydrogen atom at 298 K for C° = 1 M). While the values of k_nH_ calculated for X (0.501 M^0.1^) and Y (0.501 M^0.05^) have the same numerical value, it is incorrect to equate them because their dimensions differ, as reflected by the difference in their respective units. If K_D_ is expressed in millimolar units, the numerical values of k_nH_ for X (1 mM^0.1^) and Y (0.708 mM^0.05^) are no longer identical.

The need to properly account for stoichiometry is one reason that the contribution of an intermolecular contact (or a substructure) to affinity is not an experimental observable [8] although this appears to be the case even when stoichiometry is not an issue [28]. Some of the entropy of binding results from molecular interactions (e.g., between water molecules) that are non-local with respect to protein–ligand contacts. Some contributions to binding enthalpy, such as the enthalpic penalties associated with ligand and target adopting their bound conformations are also inherently non-local. A less obvious example of a non-local effect would be substitution at one position of a molecular structure preventing a substituent at another position from forming optimal interactions with the target. When interpreting binding thermodynamics in terms of molecular interactions, it should always be kept in mind that intermolecular contacts (e.g., between unbound ligand and solvent) that are not present in the protein–ligand complex also influence ΔH and ΔS°.

Target interaction potential (TIP) can be a helpful concept when considering association of ligands with their targets. TIP takes account of both the nature of the interactions (e.g., hydrogen bonds) and the fact that ligand-target association takes place in an aqueous environment. Hotspots [56] on the molecular surface of a target can be seen as regions of high TIP while ligandability [57] is determined both by the magnitude of TIP and the extent to which it can be exploited. An ability to reversibly form covalent bonds (e.g., catalytic cysteine thiol or a protein-bound metal cation such as zinc) with ligands would generally be associated with high TIP as would depletion [58] of water from a binding pocket or the “frustrated” hydration [43] resulting from the overlap of solvation spheres of adjacent hydrogen bond donors (or acceptors). A key challenge in drug design is to determine whether inadequate affinity is due to low TIP (i.e., target is the problem) or underexploited TIP (i.e., compound is the problem).

## Perception of efficiency varies with concentration unit

Although the implications for LE of the dependence of ΔG° on C° were first highlighted by Zhou and Gilson [7], they have been overlooked by LE advocates. For example, Murray et al. [59] discussed the validity of LE but demonstrated no awareness of the relevance of the dependence of ΔG° on C° or the fact that the logarithm function cannot take dimensioned arguments [9]. A *Future Medicinal Chemistry* editorial [60] claimed that *“Ligand efficiency validated fragment*-*based design”* while reassuring its readers that *“There is no need to become overly concerned with noisy arguments for or against ligand efficiency metrics being exchanged in the literature.”* However, this editorial [60] neither makes reference to criticism [7] of LE made in 2009 nor does it address the implications [8] of the nontrivial dependence of the metric on what is an entirely arbitrary concentration unit.

Some of the problems that result from using LE as a design metric can be seen more clearly if it is expressed using a base 10 logarithm and without energy units:

11 $$ \eta_{bind} = - \left( {\frac{1}{{N_{nH} }}} \right) \times \log_{10} \left( {\frac{{K_{D} }}{C^\circ }} \right) = \frac{\Delta g}{{RT\ln \left( {10} \right)}} $$

The quantity η_bind_ is related to Δg by a multiplicative factor of RTln(10) that is independent of C° and therefore both quantities respond in an identical manner to a change in C°. One rationale for using η_bind_ is that drug discovery scientists typically use pIC_50_ or pK_D_ rather than ΔG° in SAR analysis. The quantity η_bind_ is also related to ligand efficiency by atomic number (LEAN) [61] that is calculated by scaling pIC_50_ by N_nH_. Unlike LEAN, η_bind_ is a function of C° and can also be written as η_bind_(C°) to emphasize this. Although standard state conventions do not apply to potency measures such as IC_50_ and EC_50_, which are usually quoted in µM or nM, potency must still be scaled by a concentration value for the logarithm calculation because the logarithm function is not defined for dimensioned quantities [9]. Using η_bind_ rather than ΔG° reinforces the point that the problems associated with LE are due to the mathematical behavior of the logarithm function. While the use of a concentration unit other than 1 M to define LE is unusual, there certainly is precedent for doing so. For example, minimum inhibitory concentration, used to define antibacterial efficiency [62], was scaled by mg/ml in order that the logarithm could be calculated.

Table 1 illustrates how a change in C° alters the perception of efficiency. The hypothetical set of three compounds consists of a fragment hit (N_nH_ = 10; K_D_ = 1 mM), a lead (N_nH_ = 20; K_D_ = 1 µM) and a clinical candidate (N_nH_ = 30; K_D_ = 1 nM) that would usually be regarded as equally ligand-efficient (Δg = 0.4 kcal/mol per non-hydrogen atom at 298 K for C° = 1 M) [8]. An identical η_bind_ value of 0.3 per non-hydrogen atom is indeed calculated for all three compounds when C° is set to the conventional value of 1 M. Using a value of 0.1 M for C° leads to the conclusion that the clinical candidate is more ligand-efficient than the fragment but, if C° is set to 10 M, we come to the opposite conclusion. Given that rankings of compounds can change with C° and that there appears to be no way of objectively selecting a value of C° for these calculations, neither η_bind_ nor Δg would appear to be fit for the purpose of assessing performance, potential or quality of compounds in drug discovery projects. The same criticism can be made of BEI [2], LEAN [61], LLE_AT_ [3] and LELP [3].

**Table 1:** Dependence of ligand efficiency on C°

N_nH_^b^	K_D_/M^c^	η_bind_ (C°)^a^		
		C° = 0.1 M	C° = 1 M	C° = 10 M
10	10^−3^	0.20	0.30	0.40
20	10^−6^	0.25	0.30	0.35
30	10^−9^	0.27	0.30	0.33

^a^Ligand efficiency defined in (11)

^b^Number of non-hydrogen atoms in molecular structure

^c^Dissociation constant for protein–ligand complex

The change in perception of efficiency that results from a change in C° shows that neither η_bind_ nor Δg has thermodynamic significance. A necessary, but not sufficient, condition for validity of thermodynamic analysis is that conclusions drawn from the analysis cannot depend on the choice of C°. Although C° is an integral component of the framework of solution thermodynamics, it can also be seen simply as a unit used to express affinity so that a logarithm can be calculated for K_D_. A physical quantity that is expressed in different units is still the same quantity. If perception changes when a quantity is expressed using a different unit then neither the change in perception nor the quantity itself can be regarded as physically meaningful. Provided that C° is known, − log(K_D_/C°) is physically meaningful and the effect of a change in C° is both constant and calculable. In contrast, knowing values of η_bind_ and C° does not allow us to calculate the η_bind_ value corresponding to another value of C°. Furthermore, the results presented in Table 1 show that a single η_bind_ value can transform to more than one η_bind_ value in response to a change in C°.

The change in perception resulting from a change of unit raises the question of whether or not LE can accurately be described as a metric. The defining characteristic of a metric is that it measures and it is necessary to state clearly what a quantity measures if claiming that the quantity is a metric. While units are essential for measurement, a valid and credible framework for measurement must allow for quantities to be expressed in different units (e.g. µM and nM). For example, readers might consider their likely responses to a hypothetical report that the space group for a crystal structure differed according to whether unit cell parameters were expressed in Ångstrom or in nanometer units. There are two reasons that LE should not be considered to be a metric. First, it is not clear what LE measures since neither the extent to which molecular interactions are optimal nor interaction quality are experimental observables. Second, LE has a unit (1 M) built into it and perception of efficiency is altered (Table 1) when another concentration unit is used. It would actually be more accurate to describe LE as a simple predictor of potency in cell-based assays or of in vivo activity that, like property forecast index [49], has neither been optimized nor validated for prediction.

**Fig. 1:**
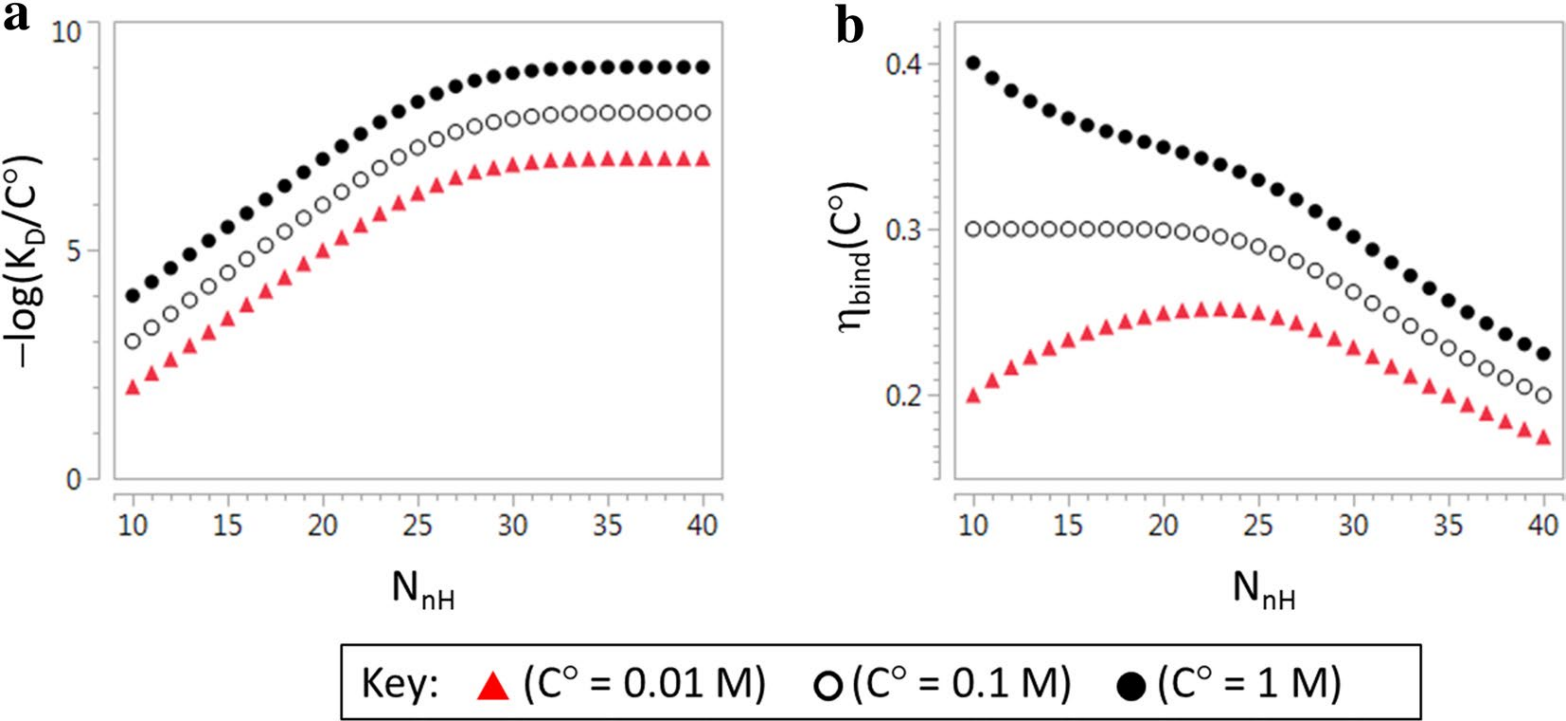
**a** Hypothetical response of affinity to molecular size for plotted for three different C° values, **b** plot of LE (η_bind_) calculated from the response of affinity to molecular size using three different C° values

Figure 1 illustrates the impact of the dependence of η_bind_ on C° in an alternative manner to Table 1. Figure 1a shows a hypothetical response of affinity to N_nH_ that has been constructed to have a linear region (slope = 0.3 per non-hydrogen atom) at low N_nH_ and a plateau at high N_nH_. The response is plotted for three different values (1 M; 0.1 M; 0.01 M) of C° and this shows how expressing affinity in a different unit shifts the entire response by a constant amount without affecting the shape of the response. In contrast, using a different unit to express η_bind_ actually changes the shape of the response of η_bind_ to C°. Furthermore, transformation of affinity to η_bind_ typically makes it more difficult to perceive the linear and plateau regions of the response of affinity to N_nH_. The results presented in Fig. 1 highlight the importance of observing relationships between affinity and molecular size directly.

LE is used to specify affinity cutoffs as a function of molecular size and a Δg value of 0.3 kcal/mol per non-hydrogen atom has been suggested [3]. Specification of affinity cutoffs in this manner forces the line defining acceptable affinity to intersect the affinity axis at a point corresponding to a K_D_ value of 1 M. This causes considerable difficulties when the range in N_nH_ is large as is the case for beyond rule of 5 (bRo5) [63] compounds. The minimum Δg value of 0.12 kcal/mol per non-hydrogen atom recommended [63] for bRo5 compounds can be translated (C° = 1 M; T = 300 K) to pK_D_ values corresponding to the lower (700 Da; N_nH_ ≈ 50) and upper (3000 Da; N_nH_ ≈ 214) limits for bRo5 space. The lower (pK_D_ = 4.4) of these two values would not appear to be a useful design criterion while the higher value (pK_D_ = 18.7) would not generally be measurable. In general, affinity acceptability thresholds should be specified directly and LE should only be used for this purpose if supported by the data.

LE was introduced [1] with the claim that it was useful but it is rarely, if ever, shown to be predictive of pharmaceutically-relevant behavior. As such, the utility of LE as a design metric hinges on it being meaningful and this places a burden of proof on those who advocate the use of LE to demonstrate that their choice of unit is universally appropriate. The importance of physicochemical properties is widely accepted in drug design and many medicinal chemists would regard it as routine to monitor progress in projects by plotting potency against molecular size or lipophilicity. A critique of LE metrics actually emphasized the importance of modeling relationships between affinity and risk factors for compounds of interest [8]. However, a depiction [6] of an optimization path for a project that has achieved a satisfactory endpoint is not direct evidence that consideration of molecular size or lipophilicity made a significant contribution toward achieving that endpoint. Furthermore, explicit consideration of lipophilicity and molecular size in design does not mean that efficiency metrics were actually used for this purpose. Design decisions in lead optimization are typically supported by assays for a range of properties such as solubility, permeability, metabolic stability and off-target activity as well as pharmacokinetic studies. This makes it difficult to assess the extent to which efficiency metrics have actually been used to make decisions in specific projects, especially given the proprietary nature of much project-related data.

## Ligand efficiency and fragment-based design

LE features prominently in the literature of fragment-based lead discovery (FBLD) [64–69] to the extent that it is sometimes presented as an important rationale for screening fragments. For example, it has been claimed [65] that *“fragment hits typically possess high ‘ligand efficiency’ (binding affinity per heavy atom) and so are highly suitable for optimization into clinical candidates with good drug*-*like properties”.* It has been asserted [31] that *“fragment hits form high*-*quality interactions with the target”* although it is not clear if interaction quality involves aesthetic aspects in addition to the physical forces more usually associated with molecular recognition [26, 27]. I would argue that the rationale for screening fragments against targets of interest is actually based on two conjectures. First, chemical space can be covered most effectively by fragments because compounds of low molecular complexity [18, 21, 22] allow TIP to be explored [70–74] more efficiently and accurately. Second, a fragment that has been observed to bind to a target may be a better starting point for design than a higher affinity ligand whose greater molecular complexity prevents it from presenting molecular recognition elements to the target in an optimal manner. While proving either conjecture definitively is difficult, the success [75] of fragment-based approaches indicates that the underlying assumptions are reasonable.

The Johnson et al. [76] study examined start-finish differences in LE for a number for a number of fragment-to-lead (F2L) optimizations that had been published in 2016 and there is precedent [49] for analyzing start-finish differences for projects in this manner. Johnson et al. [76] noted that differences in LE between fragment hits and leads were not statistically significant and stated that *“in contrast to anecdotal reports that LE tends to decline during the F2L process, LE decreased during optimization for only a minority of examples”*. This analysis is repeated using Δη_bind_ values calculated for different values of C° ranging from 0.001 to 10 M and the results are summarized in Table 2. As C° increases, the leads appear to become less ligand-efficient in comparison with the fragment hits from which they had been derived and this is analogous to what is shown in Table 1. Statistically significant differences between values for fragment hits and leads are only observed if C° is 0.01 M or 0.001 M.

**Table 2:** Dependence on C° of mean changes in ligand efficiency for F2L programs surveyed in [76]

C°/M^a^	Mean Δη_bind_^b^	SE Δη_bind_^c^	Prob > |t|^d^
0.001	+ 0.087	0.012	< 0.001
0.01	+ 0.055	0.015	0.001
0.1	+ 0.024	0.018	0.20
1.0	− 0.008	0.021	0.69
10	− 0.04	0.024	0.11

^a^Standard concentration

^b^Mean change in ligand efficiency as defined in (11)

^c^Standard error in mean change in ligand efficiency as defined in (11)

^d^*P* value for matched pair t test

Comparison of LE values for fragment hits and the corresponding leads can be seen as an attempt to quantify how effectively an increase in molecular size translates to affinity gain over the course of an F2L project. This is still a valid objective even though the LE metric would appear to be unfit for this purpose. The most obvious way to do this is to scale ΔpK_D_ by ΔN_nH_ for the F2L pairs:

12 $$ \frac{{\Delta pK_{D} }}{{\Delta N_{nH} }} = \left( {\frac{1}{{N_{nH} \left[ L \right] - N_{nH} \left[ F \right]}}} \right) \times \log_{10} \left( {\frac{{K_{D} \left[ F \right]}}{{K_{D} \left[ L \right]}}} \right) $$

The quantity defined in Eq. (12) can regarded as a measure of MSE for the F2L optimization. Using ΔpK_D_ (the logarithm of a ratio of K_D_ values) eliminates the dependency on C° that makes Δη_bind_ (and Δg) unsuitable for comparison of start and end points for projects. An additional benefit is that ΔpK_D_ is likely to be relatively insensitive to the approximation of K_D_ by IC_50_. This approach to assessing optimizations has precedent and Hajduk [33] reported that a tenfold improvement in K_D_ corresponded to a mean increase in molecular weight of 64 Da (standard deviation = 18 Da) for 73 compound pairs from FBLD projects. Hajduk [33] also illustrated the benefit of observing the response of affinity to an increase in molecular size directly rather than indirectly by using the LE metric.

It can be useful to compare the changes in affinity and lipophilicity that result from structural elaboration and one way of achieving this is to offset the change in affinity by change in lipophilicity:

13 $$ \Delta pK_{D} - \Delta \log P = \log_{10} \left( {\frac{{K_{D} \left[ F \right] \times P\left[ F \right]}}{{K_{D} \left[ L \right] \times P\left[ L \right]}}} \right) $$

The quantity in Eq. (13) may be regarded as a measure of the lipophilicity efficiency for the F2L optimization and is equivalent to the change in what would be termed LLE [3, 41] or LipE [42]. It is desirable that it should be as large as possible for F2L projects. Variations of Eq. (13) can also be written using potency (e.g. pIC_50_) with a measured distribution coefficient (logD) or a predicted [77] value of logP. Where pK_D_ was not available, it has been approximated by pIC_50_ and the ClogP values reported in Johnson et al. [76] are used as the measure of lipophilicity.

**Fig. 2:**
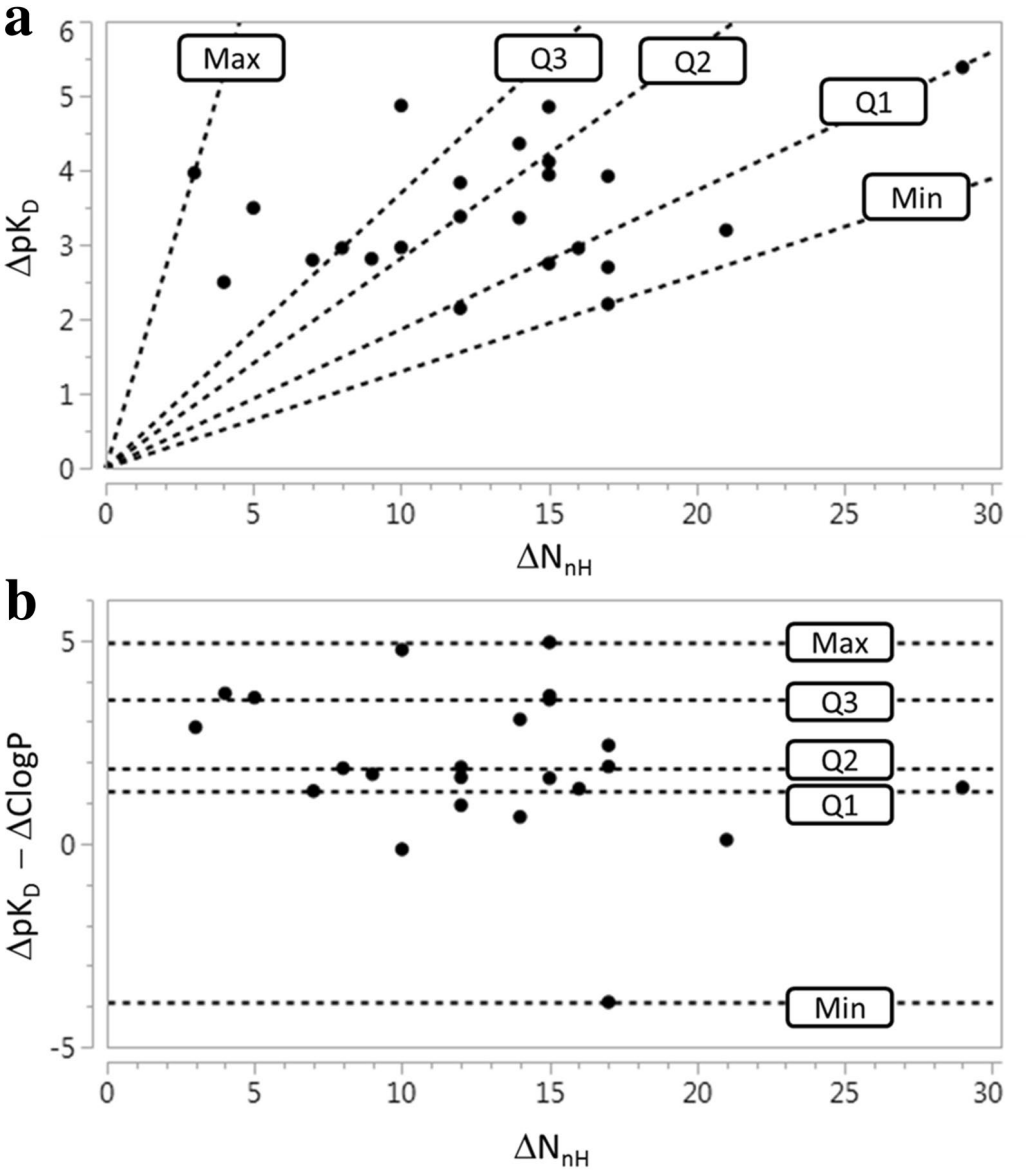
**a** Plot of ΔpK_D_ against ΔN_nH_ for F2L optimizations showing reference lines for minimum (min = 0.13), first quartile (Q1 = 0.18), median (Q2 = 0.28), third quartile (Q3 = 0.37) and maximum (max = 1.32) values of (ΔpK_D_/ΔN_nH_), **b** plot of (ΔpK_D_ − ΔClogP) against ΔN_nH_

A plot of ΔpK_D_ against ΔN_nH_ with reference lines of constant (ΔpK_D_/ΔN_nH_) is shown in Fig. 2a. This depiction is intended to map the distribution of (ΔpK_D_/ΔN_nH_) values so the reference lines are drawn to mark quartiles rather than at equally-spaced intervals. A plot of (ΔpK_D_ − ΔClogP) against ΔN_nH_ is shown in Fig. 2b with reference lines to map the distribution. Affinity gains for optimizations with zero values of (ΔpK_D_ − ΔClogP) would typically be regarded as being entirely due to increased hydrophobic contact with target. However, it is important to be aware that the octanol/water partitioning system is relatively insensitive to the presence of hydrogen bond donors [14, 43]. Large positive values of (ΔpK_D_ − ΔClogP) might be considered to be evidence that the optimization process has introduced additional polar interactions although polar functionality can still be tolerated, for example by being exposed to solvent, without actually making contact with target.

An observation that can be made about the Johnson et al. [76] analysis is that the start points for 15 of the 28 F2L projects surveyed do not appear to comply with the rule of 3 (Ro3) [78] if Ro5 [20] hydrogen bond definitions are used. This would appear to contradict the claim [75] that *“Most libraries consist of molecules that adhere to the ‘rule of three’.”* It has been suggested [22, 79] that Ro3 [78] may be overly restrictive and applying the rule would eliminate carboxylic acid bioisosteres [80] such as tetrazole [81] and N-acylsulfonamide [82] as well as the isocytosine fragment hit [83] that led to the discovery of potent β-secretase inhibitors [84]. All lead compounds surveyed in Johnson et al. [76] are of greater molecular size than the corresponding fragments but this is not the case for lipophilicity. Calculated logP values for five of the leads were lower than for the fragment hits from which they were derived, suggesting that a logP cutoff value of 3 may be overly restrictive for design of compound libraries for FBLD. The Ro5 [20] cutoff values for molecular weight (500 Da) and logP (5) were directly derived from the relevant data since they correspond to specific percentiles in the distributions observed for these quantities. However, it should not automatically be assumed that there is an analogous correspondence between the Ro3 [78] cutoff values for molecular weight (300 Da) and logP (3).

## Group efficiency

Medicinal chemists typically view SAR in terms of affinity differences resulting from structural modifications. Observation that a small structural change leads to a large change in affinity is usually informative. Group efficiency (GE) [34] is defined for the addition of a group, X, to A by scaling the value of the associated ΔΔG (*ΔG*_*X*_^*i*^ as defined in [51]) by ΔN_nH_:

14 $$ GE\left[ {A \to AX} \right] = - \left( {\frac{{\Delta \Delta G\left[ {A \to AX} \right]}}{{\Delta N_{nH} \left[ {A \to AX} \right]}}} \right) $$

The notation [X → Y] can be used to specify structural transformations and to indicate that a change in the value of a property such as ΔG°, pK_D_ or N_nH_ has been calculated by subtracting the value of the property for compound X from that for compound Y [85]. The definition of GE expresses Eq. (12) in terms of free energy rather than dissociation constant and Eq. (13) could be used in an analogous manner to specify the efficiency of substitutions from the perspective of lipophilicity. GE was stated [86] to be *“a more sensitive metric to define the quality of an added group than a comparison of the LE of the parent and newly formed compounds”* and the introduction of the metric can be seen as an attempt to address a perceived deficiency in LE. The more fundamental difference between the two metrics is that GE is independent of C° because it is defined in terms of ΔΔG. Although GE is sometimes presented as a substructural (e.g. chloro substituent) property, it is actually structural transformations (e.g. substitute hydrogen with chlorine) with which values of GE should be associated. The ΔΔG values used for calculation of GE cannot generally be interpreted as substructural contributions to affinity because summation of values of ΔΔG (ΔN = 0) cannot reproduce the dependency of ΔG° (ΔN = − 1) on C°.

**Fig. 3:**
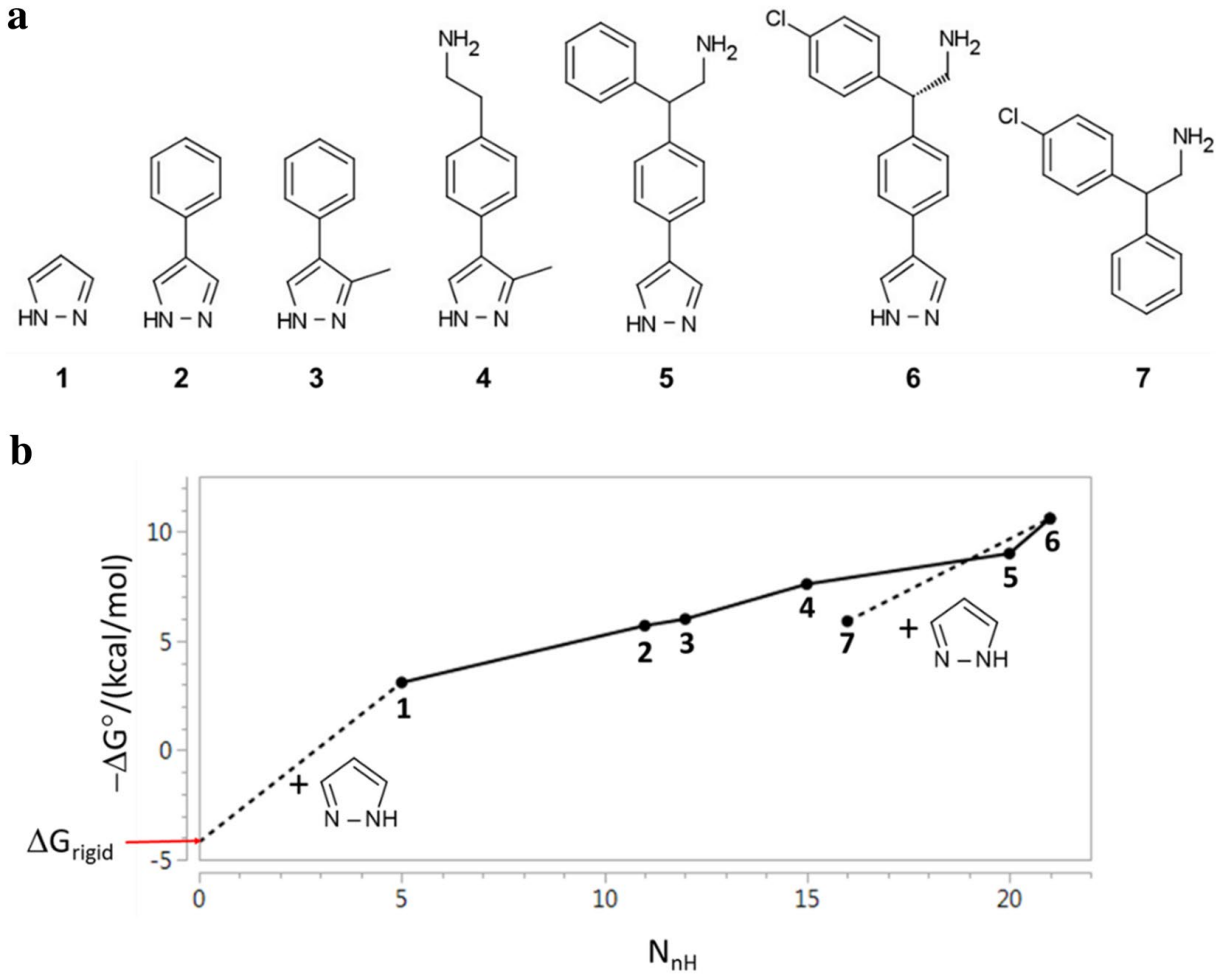
**a** Compounds described in Ref. [34], **b** plot of − ΔG° against N_nH_; broken lines indicate steps corresponding to addition of pyrazole

The GE analysis in Saxty et al. [34] was performed using data from a fragment growing project directed against protein kinase B (PKB) that is summarized in Fig. 3. The ΔG° values in Saxty et al. [34] had been calculated from IC_50_ measurements and, in one case (**1**), K_D_ estimated from crystallographic occupancy. The structural prototype **1** (N_nH_ = 5) that defines the series, is included in the data set although it was not the initial screening hit. Affinity measurements are rare for fragments of this size and are particularly valuable for mapping biophysical limits of binding. The slope of the line joining each pair of results in Fig. 3b indicates the sensitivity of affinity to an increase in molecular size for each structural transformation and in some cases this is equal to GE. Compound **5** is racemic and so [**5** → **6**] can be considered to be a composite transformation consisting of both chloro-substitution and chiral resolution. The (ΔpIC_50_/ΔN_nH_) value of 0.37 per non-hydrogen atom for [**3** → **6**] would place it on the Q3 reference line for the F2L optimizations surveyed in Johnson et al. [76].

Saxty et al. [34] reported a GE value of 1.5 kcal/mol per non-hydrogen atom for the structural prototype **1**. The substructural transformation leading to **1** poses special difficulties for ΔΔG calculation since this requires that an affinity value be assigned to a species of zero molecular size. The ΔΔG value for this transformation was derived [34] by subtracting an estimate for rigid body entropy (ΔG_rigid_ = 4.2 kcal/mol) lost on binding from the ΔG° value for **1**. The large GE value calculated for **1** is presented [34] as evidence that the interactions of the pyrazole substructure with PKB make a particularly large contribution to affinity. One interpretation of the analysis presented in Saxty et al. [34] appears to be that to be that the molecular interactions of the pyrazole substructure of **6** are assigned full credit for overcoming the penalties resulting from loss of translational and rotational entropy. This interpretation appears to be based on two assumptions. First, **1** and **6** lose identical amounts of rigid body entropy when they bind to PKB. Second, the pyrazole substructure makes identical interactions with the protein when **1** and **6** bind to PKB.

In SAR analysis, it would not be considered generally feasible to infer the importance of a substructure as a determinant of affinity using only measurements for compounds in which the substructure is conserved. Calculation of GE for **1** appears to require that a value of K_D_ be assigned to a species of zero molecular size. The value of GE derived in this manner is determined just as much by the affinity assumed for the zero molecular size species as by the affinity that is actually measured for the compound. The ΔG° values for **7** (− 5.9 kcal/mol) and **6** (− 10.6 kcal/mol) can also be used to derive a GE value of (0.9 kcal/mol per non-hydrogen atom) for the addition of the pyrazole to **6**. It is unclear why the GE value of 1.5 kcal/mol per non-hydrogen atom is preferred to the value of 0.9 kcal/mol per non-hydrogen atom that can be derived from the ΔG° values measured for **6** and **7**.

The F2L optimization reported by Saxty et al. [34] is essentially a sequence of substitutions and ΔΔG values can be associated with structural modifications in a consistent manner. Drug design frequently consists of optimization of groups at two or more substitution sites on a scaffold and non-additive [87–92] SAR needs to be considered. Free and Wilson [52] were fully aware of the problems that can result from non-additive SAR and it is not possible to assign ΔΔG values (and therefore GE values) in a consistent manner to individual structural modifications if SAR is non-additive. Subadditive SAR should be should be anticipated whenever there is a high degree of constraint in the system and might be considered to be a natural consequence of high molecular complexity [18]. Structural features likely to constrain ligand-target binding include conformational rigidity and multiple hydrogen bonds between ligand and target.

**Fig. 4:**
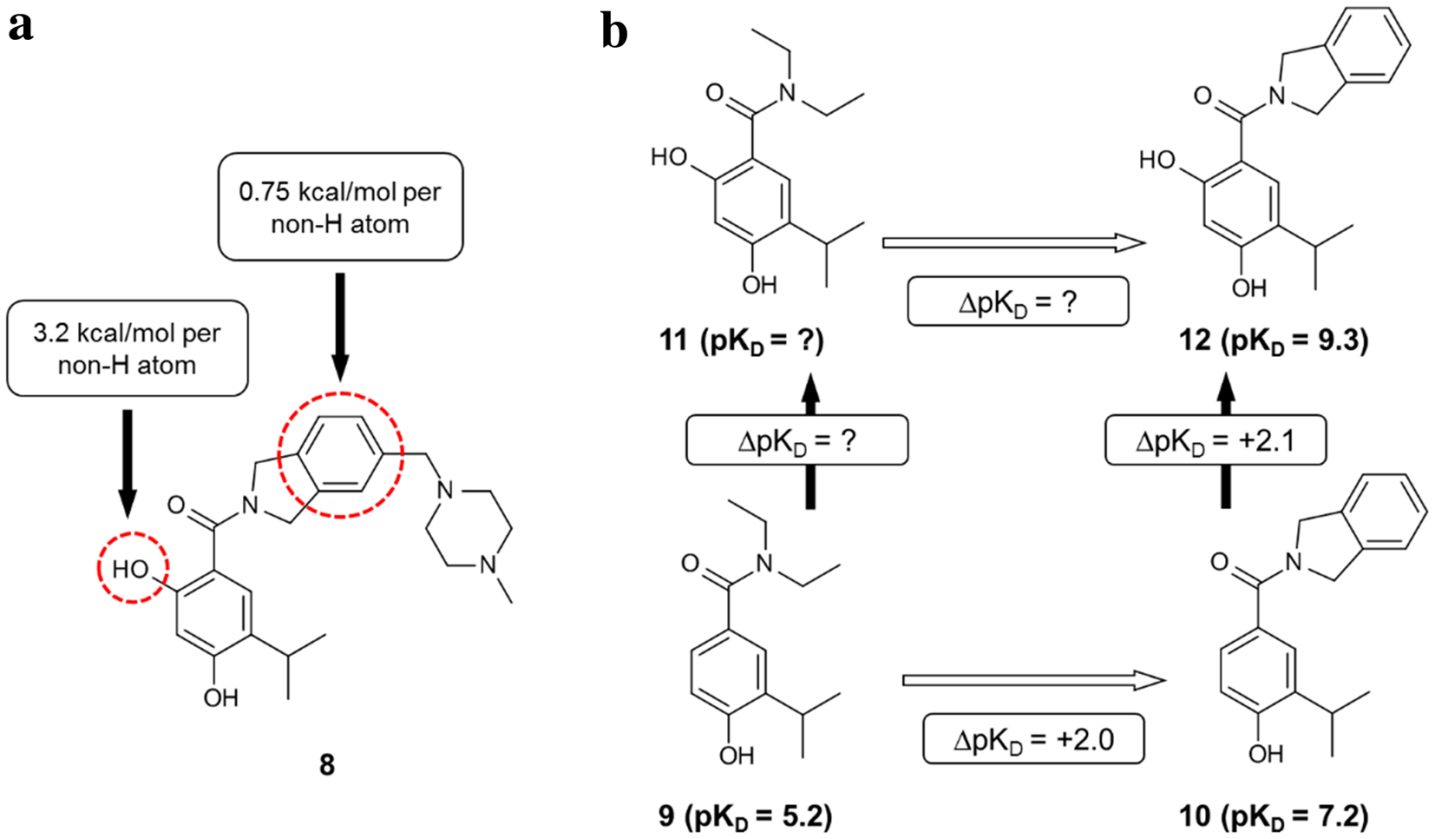
**a** Group efficiency values reported in Ref. [3] for an HSP90 inhibitor, **b** SAR of HSP90 inhibitors from Ref. [92]

Figure 4 illustrates some of the difficulties in using GE in analysis of SAR. The two GE values reported [3] for the Hsp90 inhibitor shown in Fig. 4a were derived from published SAR [93] that is summarized in Fig. 4b. The (ΔpK_D_/ΔN_nH_) value of 0.8 per non-hydrogen atom for [**9** → **12**] would place it in the top quartile of the F2L optimizations surveyed in Johnson et al. [76]. Addition of the second hydroxyl group and merging the diethylamino group with a benzene ring each appears to result in an affinity increase of two orders of magnitude. However, it is not possible to determine whether or not the SAR is additive without knowing the affinity for **11**. Graphics such as Fig. 4a cannot capture the order in which the structural transformations are carried out and, in some cases, even the starting point for the transformation is not clear. For example, it is not possible to determine from Fig. 4a whether the isoindoline in **8** had been derived from the corresponding dimethylamino, diethylamino or pyrrolidinyl group.

## Maximal affinity of ligands and fit quality

Kuntz et al. [32] explored the limits that protein structure may impose on affinity and it is also is widely regarded to have introduced the LE concept. Kuntz et al. [32] used affinity measurements against multiple targets and medicinal chemists should not automatically assume that this study is directly relevant to specific targets on which they may be working. Put another way, if a micromolar activity against a target of interest has been observed for a compound, how useful is it to know that another compound of comparable molecular size has shown picomolar affinity against another target? Sample size is an important consideration in studies of biophysical limits of affinity since the observation of maximal affinity can be regarded as a relatively rare event. How many observations of affinity against how many targets would one need to make for compounds with 20 non-hydrogen atoms in order to have a 95% chance of observing affinity within 1.4 kcal/mol of a theoretical affinity limit for compounds of this molecular size?

Kuntz et al. [32] analyzed the relationship between ΔΔG_binding_ (numerically equivalent to ΔG° for C° = 1 M) and number of non-hydrogen atoms:

If we ignore simple cations and anions, the data show a sharp improvement in binding free energy until ≈ 15 heavy atoms per molecule. The ΔΔG_binding_ binding of the tightest-binding ligands then plateaus at ≈ − 15 kcal/mol (i.e., picomolar dissociation constants). The initial slope is approximately − 1.5 kcal/mol per atom.

While the response of ΔG° to N_nH_ (-0.44 kcal/mol per non-hydrogen atom) shown in Fig. 3b is much less steep than the initial response of ΔΔG_binding_ to N_nH_ (− 1.5 kcal/mol per non-hydrogen atom) reported by Kuntz et al. [32], its linearity appears to be maintained over the entire molecular size range (5 ≤ N_nH_ ≤ 21) for the data. The findings from Kuntz et al. [32] do not appear to provide insights that would be useful for the interpretation of the results shown in Fig. 3b and this reflects the fact that affinity measured against multiple targets was used for the analysis in that study. While the ΔΔG_binding_ values used in Kuntz et al. [32] do not depend on C°, the line describing the initial response of ΔΔG_binding_ to N_nH_ was constrained to pass through the point (N_nH_ = 0; ΔΔG_binding_ = 0). Shultz noted [94] that imposition of this constraint (equivalent to assuming that K_D_ = 1 M for zero molecular size) is likely to have biased the estimate for the steepness of the initial response and others [8] subsequently made similar observations.

The response of maximal affinity to molecular size shown in Kuntz et al. [32] might be anticipated from consideration of molecular complexity [18] and it provides support for the view that additivity [87–92] in SAR decreases with molecular size. Although the choice of intercept in Kuntz et al. [32] has been criticized [8, 94], the response of maximal affinity to molecular size was modeled directly in the study. In contrast, Reynolds et al. [95] modeled the response of maximal LE to molecular size. Reynolds et al. [95] asserted that *“ligand efficiency is dependent on ligand size with smaller ligands having greater efficiencies, on average, than larger ligands”* and Murray et al. [59] repeated this assertion. As shown in Fig. 1b, the apparently greater efficiency of smaller ligands can reflect the choice of unit used to express affinity and, therefore, should be not interpreted as having any special significance.

Reynolds et al. [95] used fit quality (FQ) to normalize LE with respect to molecular size and claimed that *“the fit quality score provides a simple method for directly measuring how optimally a ligand binds relative to other ligands of any size”*. However, the results presented in Table 1 show that it is not valid to claim that LE measures how optimally a ligand binds, even to a single protein, since rankings of compounds can vary with the concentration unit in which K_D_ is expressed. Given that the degree to which a ligand binds optimally has not been shown to be an experimental observable, it would not be valid to make a claim for direct measurement even if perception of efficiency was independent of C°. FQ was introduced to address a perceived deficiency of LE and it has been stated [60] that *“LE can break down when comparing ligands of disparate size (LLE, FQ and size independent ligand efficiency [SILE] are better)”*.

The calculation of FQ involves first deriving the LE_Scale function by modelling the maximal LE as a function of N_nH_ to provide a reference for scaling LE values [95]. FQ is defined as the ratio of LE to LE_Scale which means that it is simply a ratio of ΔG° values and therefore dependent on C°. This is a separate issue from the dependence of LE on C° since the comparison between LE and LE_Scale is made using the same value of N_nH_. Although it should be possible to address the problems associated with using ΔG° ratios by using ΔΔG, there remains the issue that affinity values used for the calculation of LE and LE_scale do not generally correspond to the same protein. This means that a low value of FQ could just as plausibly be explained by low TIP of the target as by suboptimal interactions with the target.

The analysis presented in Reynolds et al. [95] can also be criticized from a general cheminformatic perspective. While the dependence of maximal binding affinity on molecular size may be of interest to drug discovery scientists, there are a number of reasons why this relationship would be better modelled directly with ΔG° (or pK_D_) as the dependent variable and N_nH_ as the independent variable. First, using affinity as the dependent variable means that there are none of the difficulties caused by the dependence of LE on C° since a change in C° simply shifts affinity by a constant amount that is independent of molecular size (see Fig. 1a). Second, it is not generally possible to assess quality of fit in a meaningful manner when fitting a quantity (e.g., pK_D_/N_nH_) that depends explicitly on the independent variable (e.g., N_nH_). This is because, to some extent, the modelling process involves fitting the independent variable to itself. Third, scaling affinity by molecular size also scales the uncertainty in the affinity by molecular size and this needs to be properly accounted for when performing regression analysis. Sheridan has debunked the suggestion that LE is inherently more predictable than affinity [96].

## Alternatives to ligand efficiency for normalization of affinity

Despite the criticisms made of the LE metric and its variants, the view that the best compounds punch above their weight is still valid. While it does not appear possible to define LE objectively in an absolute sense, the Hajduk [33] and Saxty et al. [34] studies showed that efficiency can be defined in relative terms. With appropriate data analysis, it might prove possible to establish a particular value of (ΔpK_D_/ΔN_nH_) as indicative that two compounds bind with equal efficiency.

LE was introduced [1] as a means to normalize affinity with respect to molecular size and this raises the question of whether or not meaningful normalization can be achieved without having to assume a particular value of C°. Although GE does not vary with C°, this metric is associated with structural transformations, rather than compounds, and so cannot be used to normalize affinity of compounds. To describe data as normalized would generally imply that some preliminary analysis has been performed on the data. For example, one might subtract the mean molecular weight for the fragments in a screening library from the molecular weight of each fragment. Mean-centering data in this manner makes it possible to determine at a glance whether or not a fragment in the library is larger than average.

Affinity can be normalized with respect to design risk factors such as molecular size in a manner that is analogous to mean-centering by using the trend in the data instead of the mean. This is analogous to the approach that was used in the Andrews et al. [53] study and has been outlined in previous studies [8, 43]. The response of affinity to molecular size is first modelled so that the trend in the data can be represented by the pK_D_ values predicted by the model. Affinity is then normalized by subtraction of predicted pK_D_ from the experimentally measured value (this difference is conventionally referred to as the residual):

15 $$ pK_{D} \left[ {resd} \right] = pK_{D} \left[ {expt} \right] - pK_{D} \left[ {pred} \right] $$

A large absolute value of a residual can be seen as a type of activity cliff [37, 38] and the most interesting SAR is likely to be associated with the most deviant values. A large positive residual could reflect a different type of molecular interaction while a large negative residual might be linked to the absence of a key molecular recognition element. Affinity (or potency) should always be plotted as a function of molecular size during the course of an F2L project and even a weak trend can be used to normalize data. Modelling the data in the initial stages of an F2L project may indicate the likely response of affinity to a further increase in molecular size and the greater sensitivity to structural elaboration of one fragment hit may trump the greater potency of another when setting priorities.

**Fig. 5:**
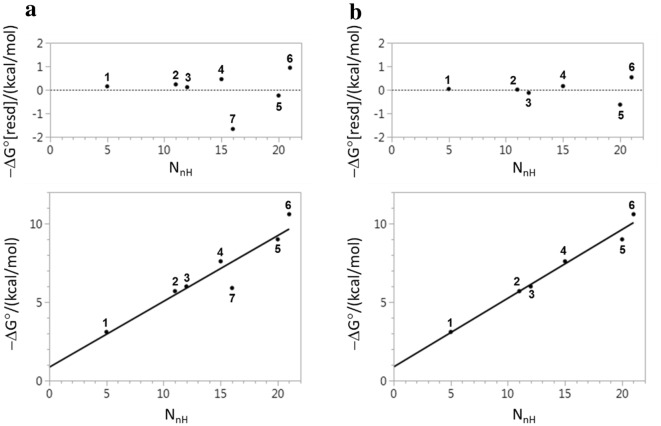
Normalization of data from reference 34 using residuals **a** data set includes **7**, **b** data set excludes **7**

This approach to normalization of affinity with respect to molecular size can be illustrated using the data from Saxty et al. [34]. The results of fitting − ΔG° to N_nH_ are shown in Fig. 5 and summarized in Table 3. The large negative residual (− 1.7 kcal/mol) for **7** shown in Fig. 5a highlights the importance of the pyrazole substructure for affinity. One advantage of analyzing the data in this manner is that all compounds are treated equivalently in the analysis so there is no need to make a special case of the structural prototype **1**.

**Table 3:** Results of fitting linear model to data from Ref. [34]

Dataset	− ΔG°/(kcal/mol) = A_0_ + (A_1_ × N_nH_)						
	N^a^	A_0_	SE A_0_^b^	A_1_	SE A_1_^c^	RMSE^d^	R^2e^
Compounds **1**–**7**	7	0.85	1.00	0.42	0.07	0.89	0.89
Compounds **1**–**6**	6	0.87	0.48	0.44	0.03	0.42	0.98

^a^Number of observations

^b^Standard error in A_0_

^c^Standard error in A_1_

^d^Root mean square error

^e^Coefficient of determination

Once it has been established that the pyrazole substructure is important for affinity, the non-pyrazole **7** can be excluded from the dataset to enable affinity to be normalized for the pyrazoles (Fig. 5b). The results in Table 3 show a very strong relationship (R^2^ = 0.98; RMSE = 0.42 kcal/mol) between ΔG° and N_nH_ and practically all the variation in ΔG° for these compounds can be explained by differences in molecular size. The two residuals of greatest magnitude correspond to **5** (− 0.6 kcal/mol) and **6** (+ 0.5 kcal/mol) and these values reflect the large GE value of 1.6 kcal/mol per non-hydrogen atom reported for the [**5** → **6**] transformation. A significant portion (≈ − 0.4 kcal/mol) of the residual for **5** can probably be explained by its racemic nature. Had the more active enantiomer of **5** been used in the analysis, the GE value for the chloro-substitution might have been 1.2 kcal/mol per non-hydrogen atom rather than 1.6 kcal/mol per non-hydrogen atom as reported in Saxty et al. [34]. Both the residual for **6** and the GE value for the [**5** → **6**] transformation highlight the importance of the chloro substituent as a molecular recognition element in this system. Given that the pyrazole ring is present in all structures, it is not possible to draw any inference about the contribution of this molecular recognition element to affinity although the excellent linear fit to the data shown in Fig. 5b is consistent with a view that structural elaboration did not compromise the hydrogen bonding between pyrazole and the hinge region of PKB.

Analyzing affinity data in this manner effectively partitions MSE for a compound into a term that characterizes the steepness of response of affinity to molecular size for the particular selection of compounds and a residual term that quantifies the extent to which the affinity of a compound beats (or is beaten by) the trend in the data. The residuals are invariant with respect to change in C° so there is no change in perception if affinity is expressed using a different concentration unit. Although residuals cannot be used to define efficiency in an absolute sense, compounds can still be ranked and there is no requirement, as is the case for analysis based on GE [34, 86], that the compounds be structurally related. Affinity can be normalized with respect to other risk factors (e.g., lipophilicity) using residuals and other properties (e.g., aqueous solubility) can be normalized in an analogous manner. When using residuals for normalization of affinity, there is no requirement that the model be either linear or univariate. This means that affinity can be normalized with respect to more than one risk factor in a single analysis.

Drug discovery scientists typically need be able to address a range of questions when interrogating project data. For example, it may be useful to focus analysis on the most active compounds in an optimization project. It is important to stress that residuals are not generated in isolation and they result from analysis that, arguably, should be performed anyway. The line fit to a plot of affinity against molecular size is likely to be a better predictor of outcome than a line that has been artificially forced to intercept the affinity axis at a point corresponding to a K_D_ value of 1 M [8]. The strength of the trend also provides an indication of how useful normalization of the data is likely to be. For example, the observation of a very weak correlation between affinity and molecular size for hits from a fragment screen may suggest that molecular size need not be accounted for when assessing the fragment hits in question. In an optimization project, a relatively weak correlation between affinity and molecular size may point to SAR that is specific to the extent that it cannot be adequately explained by molecular size alone.

## Conclusions

LE has been discussed in depth from a physicochemical perspective in this study and the difficulty of interpreting affinity in terms of molecular interactions was highlighted. The nontrivial dependency of LE on the concentration unit in which affinity is expressed means that LE has no physical significance and, strictly, should not even be considered to be a metric. As such, LE is unsuitable for ranking compounds, setting acceptability thresholds for affinity and modeling relationships between affinity and molecular size. While it does not appear to be possible to quantify absolute efficiency of binding for compounds in an objective manner, efficiency can still be defined in a relative manner by scaling affinity differences by the corresponding molecular size differences.

## Abbreviations

C°: standard concentration
FBLD: fragment-based lead discovery
FQ: fit quality
F2L: fragment-to-lead
GE: group efficiency
IC_50_: half maximal inhibitory concentration
K_D_: dissociation constant
LE: ligand efficiency
LE_Scale: estimate for maximal ligand efficiency as function of N_nH_
LipE: lipophilic efficiency
LLE: ligand lipophilicity efficiency or lipophilic ligand efficiency
logD: base 10 logarithm of octanol/water distribution coefficient
logP: base 10 logarithm of octanol/water partition coefficient
MSE: molecular size efficiency
N_nH_: number of non-hydrogen atoms in a molecular structure
P: octanol/water partition coefficient
pIC_50_: − log_10_(IC_50_/M)
pK_D_: − log_10_(K_D_/M)
pK_D_[expt]: experimentally measured pK_D_
pK_D_[pred]: value of pK_D_ predicted by model
pK_D_[resd]: residual pK_D_
R: gas constant
Ro3: rule of 3
Ro5: rule of 5
T: thermodynamic temperature
TIP: target interaction potential
Δg: ligand efficiency calculated from standard free energy of binding
ΔG°: standard free energy of binding
ΔN: change in number of chemical species
η_bind_: ligand efficiency calculated from logarithmically expressed K_D_ without energy units
